# A Biopsychosocial Model Predicting Myocardial Infarction

**DOI:** 10.3390/jcm12175715

**Published:** 2023-09-01

**Authors:** José M. Tomás, Amparo Oliver, Zaira Torres, Janhavi Parker, Elena Marques-Sule, Trinidad Sentandreu-Mañó

**Affiliations:** 1 Department of Methodology for the Behavioral Sciences, University of Valencia, 46010 Valencia, Spain; tomasjm@uv.es (J.M.T.); amparo.oliver@uv.es (A.O.); 2 SMBT Institute of Medical Sciences and Research Centre, Nashik 422403, Maharashtra, India; janhaviparker@gmail.com; 3 Department of Physiotherapy, University of Valencia, 46010 Valencia, Spain; elena.marques@uv.es (E.M.-S.); trinidad.sentandreu@uv.es (T.S.-M.)

**Keywords:** biopsychosocial approach, frailty, coronary artery disease, cardiovascular disease, SHARE survey, aging

## Abstract

Myocardial infarction is one of the main causes of death, and cardiovascular risk factors (CVRFs) are always considered when studying it. However, although it is known that other social and psychological variables, and especially frailty, can increase the risk of infarction, their simultaneous effect has not been extensively studied. This study is based on data from the SHARE project (latest wave, Wave 8), with a representative sample of 46,498 participants aged 50 or older (M = 70.40, SD = 9.33), of whom 57.4% were females. Statistical analyses included a full structural equation model that predicts 27% of infarction occurrence and evidences the significant effect of well-being, depression, and social connectedness on frailty. Frailty, in turn, explains 15.5% of the variability of CVRFs. This work supports the need to study these physical, social, and mental health factors together to intervene on frailty and, in turn, improve cardiovascular outcomes.

## 1. Introduction

Myocardial infarction is caused by decreased or complete cessation of blood flow to a portion of the myocardium, which can be silent and go undetected, or it could be a catastrophic event leading to hemodynamic deterioration and sudden death [1]. Cardiovascular diseases (CVD) are the leading cause of worldwide death and a significant contributor to disability [2]. An estimated 17.8 million people died from a cardiovascular disease in 2017 and, of these deaths, 84.9% were due to ischemic heart disease and stroke [3]. Each year, myocardial infarction affects over three million individuals, underscoring its significant clinical impact [4].

A substantial body of knowledge related to its biological underpinnings has been accumulated. The intricate interplay of genetic predispositions, lipid profiles, inflammatory markers, hemodynamic factors, and the decline of sex hormones have been previously discussed [5,6,7,8]. Cardiovascular risk factors such as high blood pressure and dyslipidemia, among others, are considered in the development of myocardial infarction [5,6]. Age is an independent nonmodifiable cardiovascular risk factor that may be compounded by other additional modifiable factors, including frailty and diabetes, which are known to complicate and enhance cardiovascular risk factors associated with the onset of advanced age [6]. Understanding and identifying the cardiovascular risk factors plays a crucial role in the prevention and development of CVD such as myocardial infarction, so specific strategies can be used to mitigate the impact of these risk factors on its development [9]. In addition to medication and diet supplementation, different approaches aimed at controlling these risk factors related to lifestyle modifications, such as a healthy weight and regular exercise, have shown their protective effect [7], but little is known about the role that frailty and psychosocial factors play on the influence of these cardiovascular risk factors in people who have suffered a myocardial infarction.

Frailty is a complex age-related clinical state characterized by a decline in the physiological capacity of various organ systems. This results in an increased vulnerability to minor stressors that can increase the risk for developing adverse health-related outcomes such as dependence or death [10]. Although there is currently no consensus on its definition, one of the most accepted constructs is the Fried’s phenotype. According to the Fried criteria, the frailty phenotype is defined as the presence of at least three of the following conditions: unintentional weight loss, self-reported exhaustion, slow gait speed, low energy expenditure, and weak grip strength [11]. This approach underlines physiological and metabolic changes, which may be interconnected, and which are responsible for driving progressive physical impairments that lead to a decline in functional capacity [12,13].

Recent research highlights frailty as a potential risk factor for CVD. A recent meta-analysis found a significant link between frailty and prefrailty stages and a higher risk of incident CVD over around 4.4 years, even after accounting for confounders like age, gender, and cardiovascular risk factors [14]. Moreover, the presence of frailty syndrome increases the risk for faster onset of any type of CVD (regardless of classical cardiovascular risk factors) and multiplies by approximately fourfold the risk of death from cardiovascular causes [15]. While the intricate pathways connecting frailty and CVD involve complex mechanisms, the shared pathological background, particularly inflammatory paradigms, might contribute to their association [14,16]. However, the precise underlying mechanism remains mostly hypothetical. One plausible theoretical framework connecting frailty and CVD is “inflammageing” characterized by elevated proinflammatory markers from aging tissues [17]. Current evidence shows that high levels of inflammatory markers in blood are linked to depression, muscle weakness, reduced muscle mass, poor function, and limited mobility, all posing risks for frailty and cardiac disease [18,19]. Oxidative stress is another theory of interest. Frail individuals often exhibit heightened levels of oxidative stress biomarkers [19,20], which is connected to chronic inflammation and progression to conditions like CVD [6,21], suggesting a role in the pathogenesis of both conditions [22]. Another conceptual framework involves cellular senescence triggering systemic inflammation. Among studied organ systems, cardiovascular dysfunction displays the strongest link with frailty [23]. In this context, frailty could predispose heart tissue to cellular senescence changes and tissue damage [24,25].

In the context of myocardial infarction, previous studies have highlighted the significance of frailty as a crucial prognostic indicator for adverse outcomes like mortality, bleeding, and rehospitalization, among others, following an acute myocardial infarction [26,27,28]. However, evidence connecting frailty and myocardial infarction is limited [29], and a knowledge gap persists regarding frailty and its associated cardiovascular risk factors. Frailty is common in hypertensive individuals, estimated at 72% prevalence in frail people and 14% in hypertensive patients [30]. The relationship between lipid profiles and frailty status remains controversial. Sergi et al. [31] reported a marginally significant increase in HDL concentration in prefrail individuals compared to non-frail ones, while the ELSA study found no differences in total cholesterol or HDL levels between frail and non-frail subjects [32]. Divergent findings also exist concerning the link between frailty and diabetes. The British Regional Heart Study revealed a high prevalence of diabetes in frail subjects but failed to establish a clear connection between impaired fasting glucose and both frailty and prefrailty syndrome, regardless of the presence of established CVD [33]. Conversely, the longitudinal ELSA Study found those diagnosed with diabetes at baseline more prone to develop physical frailty [32].

Research emphasizes the impact of psychosocial factors on physical health [34,35,36]. Illustrating this link, psychological well-being in later life may protect against physical frailty [37], while older adults with depression face a fourfold risk of frailty [34]. Recent studies have focused on associations between frailty and psychosocial factors such as loneliness and/or social isolation. Loneliness refers to subjectively perceived deficits in social support or social connections, whereas social isolation is a more objective measure of the lack of social contact [38]. A recent study has shown that older people are more likely to become frail if they have high levels of social isolation or loneliness [39]. Also, other studies [40,41] demonstrate a robust bidirectional relationship between frailty and the psychosocial indicators, thereby suggesting the need to delve deeper into these areas. These psychological factors present a higher risk to cardiovascular health in frail individuals. For example, social connectedness seems to diminish blood pressure reactivity [42]. In addition, loneliness has been shown to affect neuroendocrine function, leading to poorer cardiovascular health and biological function in frail individuals [43,44]. Additionally, the research to date has indicated a strong reciprocal relationship between depressive symptoms and frailty [34]. This holds across different study designs, regions, gender, and confounder variables used, but the extent, type, and mechanism underlying this relationship have yet to be deciphered [45]. Lastly, multidimensional frailty was associated with a higher risk of mortality and significantly lower quality of life [46]. In addition, psychosocial factors such as depression and stress have also been established as cardiovascular risk factors [47,48,49]. Specifically, the presence of psychosocial stressors has been associated with an increased risk of acute myocardial infarction [49]. In this line, among patients with post-traumatic stress disorder and high prevalence of depression, frailty estimates were 39.7%, and 42% of the patients had coronary artery disease [50]. Additionally, major depression has been associated with coronary artery disease in a large-scale meta-analysis [51].

Hence, the main objective of this research was to investigate the relationship between frailty, psychosocial factors, and myocardial infarction, establishing a mediational biopsychosocial model in which several background variables affect psychosocial conditions which may exacerbate frailty conditions and cardiovascular risks; these background variables, in turn, increase the likelihood of suffering a heart attack. This is a predictive model; no assumption of causal effects is made, although the model tests the compatibility (fit) of the theoretical process with the data. Also, we aim to study the occurrence of myocardial infarction from a holistic and psychosocial approach and to address the relationship between frailty and cardiovascular risk factors, thereby evaluating frailty through a psychosocial lens. This will improve the existing information since the simultaneous effect all these variables on cardiovascular risk factors and myocardial infarction has not been extensively studied.

## 2. Materials and Methods

### 2.1. Sample and Procedure

The data for this study came from the 8th wave of the Survey of Health, Ageing and Retirement in Europe (SHARE). SHARE is a longitudinal study that started in 2004 and includes an update of the data every 2 years. For this study, we employed Wave 8, the most recent. For Wave 8, the collection of the data took place largely synchronously across countries between the end of October and the beginning of November 2019. SHARE has a probability-based method aimed at populations aged 50 or more who have their regular domicile in some of the countries included in the SHARE project (26 European countries and Israel). SHARE’s sampling protocol consists of 4 steps. First, each participating country defines a sampling frame and sampling procedure. Next, sample information is collected and processed in each country, resulting in a preliminary sample file. These files are then reviewed and approved by international coordinators at SHARE headquarters. Finally, the sample files are entered into the computer system and merged with the address data [52]. The SHARE data are collected face-to-face via computer-assisted personal interviewing (CAPI). More information about the SHARE survey design is available elsewhere [53,54].

The Survey of Health, Ageing and Retirement in Europe (SHARE) project procedures conform to international ethical standards. SHARE is guided by international research ethics principles such as the Respect Code of Practice for Socio-Economic Research, and Wave 8 was approved by the ethics council of the Max Planck Society in Munich (Approval Code: 2021_24). In addition, SHARE country implementations were reviewed and approved by the respective ethics committees or institutional review boards. Participation in SHARE was voluntary, anonymous, and informed. The collection of SHARE data and their use are subject to the European General Data Protection Regulation. SHARE data can only be used for scientific purposes and are protected by the German Federal Statistics Act and the German Federal Data Protection Law.

As exclusion criteria, we only included in our sample people aged 50 years or more, since SHARE also includes the current partners of the participants living in the same household, regardless of their age. In addition, SHARE also excludes people who do not speak the language of the country or who were incarcerated, hospitalized, or out of the country during the sample period.

The present study involved 46,498 participants aged between 50 and 103 years old (M = 70.40, SD = 9.33), of whom 19,826 (42.6%) were males and 26,672 (57.4%) were males. Many of them were either married (65.4%), widowed (18.8%), or divorced (8.1%), while the rest (7.7%) presented another marital status. It is important to bear in mind the rationale behind this specific sample size. The SHARE survey is designed to produce representative samples at each wave and for each participant country. Therefore, to achieve this objective, each country uses its own sampling scheme to obtain a representative sample and, accordingly, produces valid and generalizable conclusions for all participating countries. Table 1 also showed the description of the other variables included in the study.

### 2.2. Instruments

Frailty assessment was performed by the SHARE-Frailty Index (SHARE-FI) [55,56], based on the Fried criteria, and addressed frailty with five measures: (1) self-perceived fatigue; (2) diminution of appetite; (3) lack of energy and slowness employing the dichotomous items “In the last month, have you had too little energy to do the things you wanted to do?”, “Have you been eating more or less than usual?”, and “Because of a health problem, do you have difficulty walking 100 m or climbing one flight of stairs without resting in the last 3 months?”; (4) physical activity with an ordinal variable “How often do you engage in activities that require a moderate level of energy such as cleaning the car, or doing a walk?” answered with a 4-point Likert scale between 1 (more than once a week) to 4 (hardly ever or never); and (5) handgrip strength, measured with a dynamometer.

Myocardial infarction was measured with the question “Have you ever had a heart attack?” (yes/no). Additionally, several cardiovascular risk factors were assessed with being diagnosed (yes/no) with high blood pressure or hypertension, high cholesterol, and diabetes or high blood sugar.

Depressive symptoms were measured with several indicators of the EURO-D scale [57]. These were depressed mood (“In the last month, have you been sad or depressed?”), pessimism (“What are your hopes for the future?”), suicidality (“In the last month, have you felt that you would rather be dead?”), guilt (“Do you tend to blame yourself or feel guilty about anything?”), sleep (“Have you had trouble sleeping recently?”), (lack of) interest (“In the last month, what is your interest in things?”), irritability (“Have you been irritable recently?”), (lack of) concentration (“Can you concentrate on something you read?”), (lack of) enjoyment (“What have you enjoyed doing recently?”), and tearfulness (“In the last month, have you cried at all?”). Response scale was dichotomous, with 0 indicating absence and 1 indicating presence. Two items that are part of the depression scale (fatigue and appetite) are also part of the frailty index measure. To avoid overlapping in the analysis, we removed these items from the depression scale. The psychometric properties of this scale have been proved in recent validations [58,59], and the internal consistency of the scale was α = 0.66, ω = 0.67 in this analysis.

Loneliness was assessed with the UCLA scale [60], which contains 3 items related to the frequency of feeling a lack of companionship (“How much of the time do you feel you lack companionship?”), feeling excluded (“How much of the time do you feel left out?”), and feeling isolated (“How much of the time do you feel isolated from others?”). Responses ranged from 1 (hardly ever or never) to 3 (often), with a maximum of 9. This scale has been previously validated and showed good psychometric properties [61]; for this study, the scale had reliability estimates of α = 0.76 and ω = 0.76.

Quality of life was measured with the CASP-12 [62]. It included 4 domains: control (e.g., “How often do you think your age prevents you from doing the things you would like to do?”), autonomy (e.g., “How often do you think that you can do the things that you want to do?”), self-realization (e.g., “How often do you feel full of energy these days?”), and pleasure (e.g., “How often do you look forward to each day?”), which can be combined in a total quality of life score. The scale is composed of 12 items answered in a 4-point Likert scale ranging from 1 (never) to 4 (often). Summing all item scores, a total score of quality of life is obtained, ranging from 12 to 48, with higher values indicating better quality of life. The scale has been validated in multiple studies [63,64]. Also, the CASP-12 score had a good level of internal consistency in this analysis: α = 0.83 and ω= 0.83.

As a measure of social isolation, we used the Social Connectedness scale. It combines the following aspects: network size, proximity (number of social network members living within 25 km), frequency of contact (number of cited persons with weekly or more contact), emotional closeness (number of cited persons with very or extremely close emotional ties), and diversity (the different types of relationships in the network), with lower values reflecting higher social isolation. The transformed values of the scale ranged from 0 to 4. More details about how to generate this scale could be consulted at [65]. This scale showed good performance in the initial study that developed it [66] and in other studies in which it has been used [67], and it had adequate internal consistency (α = 0.89, ω = 0.90).

Furthermore, several socioeconomic control variables were considered to minimize confounding effects including age, gender (which was dichotomous (female = 1, male = 0)); and economic status, measured with the question “Thinking of your household’s total monthly income, would you say that your household is able to make ends meet?” These questions were answered in a 4-point Likert scale ranging from 1 (with great difficulty) to 4 (easily).

### 2.3. Statistical Analyses

For the purposes of this study, a full structural equation model (SEM) was estimated using MPlus 8.7 [68] to test a model relating social and psychological variables as antecedents of frailty and cardiovascular risk factors and myocardial infarction as consequences. SEM models were used to relate variables in a multivariate context, so this procedure enables the analysis of latent variables or factors (where several indicators are used to measure the construct of interest), such as frailty and cardiovascular risk factors, without random measurement errors and, simultaneously, their predictive power to explain the myocardial infarction and their relationships. We used weighted least squares means, and variance adjusted (WLSMV) as the method of estimation. Among the methods of estimation available in the SEM framework, this is particularly adequate for non-normal and ordinal data [69]. A key aspect of any structural model is assessment of model fit, or adjustment between the data and the theoretical model proposed. Several authors, as summarized in Kline [70], have proposed that no single measure of fit can offer a clear-cut solution to model assessment, and there is consensus that several fit indices should be considered conjointly to assess good model fit. Specifically, good model fit can be declared when the Comparative Fit Index (CFI) is at least 0.90, and the root-mean-square error of approximation (RMSEA) and standardized root mean square residual (SRMR) are below 0.08 [71]. These 3 fit indices seemed to work well with these cut-offs in a simulation study, especially when full structural equation models are employed [71]. Given that there are a certain number of missing data in Wave 8 for the variables in the model, missing data were imputed in SPSS with the expectation maximization (EM) algorithm, and the model produced estimations with imputed data and without imputed data. Results in both datasets were almost identical, and, therefore, the results without imputed data will be reported. Given that SHARE is a probabilistic survey that guarantees the samples are representative of their respective populations, the generalizability of the findings obtained from the SEM analysis is also guaranteed. Parameter estimates will be offered in standardized metric. Standardized estimates offer the expected change in the outcome in standard deviations when the predictor changes in 1 standard deviation. These standardized estimates allow for relative comparisons among estimates. Acock [72] argues that they can be interpreted as: β < 0.2 = weak effect, 0.2 < β < 0.5 = moderate, and β > 0.5 = strong effect.

## 3. Results

Table 1 shows the descriptive statistics of all observed variables involved in the model. For quantitative and semiquantitative variables, mean and standard deviations are shown, whereas for categorical variables, percentages of all categories are presented.

**Figure 1:**
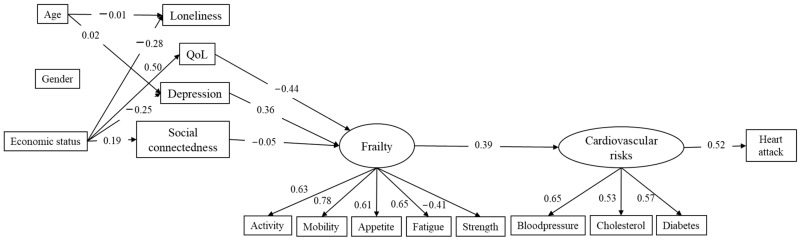
Standardized parameter estimates of the structural model to predict heart attack. Notes: only statistically significant estimates (*p* < 0.05) are shown for the sake of clarity; correlations among antecedents and among psychosocial factors not shown for clarity.

The full structural model is presented in Figure 1. From a statistical point of view, the variables age, gender, economic status, loneliness, quality of life, depression, social connectedness, and heart attack are treated as observed single indicators, while frailty and cardiovascular risks are modeled as factors (latent variables). Frailty is tapped with five observed indicators, while there are three indicators of cardiovascular risk. From the theoretical point of view, the model posits age, gender, and economic status in the household as background variables and potential predictors of the psychosocial variables (quality of life, depressive symptoms, loneliness, and social connectedness). Heart attack is the outcome to predict, while cardiovascular risks, frailty, and psychosocial conditions are mediators. These psychosocial variables (loneliness, QoL, depression, and social connectedness) are assumed to affect frailty status which, in turn, affects the likelihood of cardiovascular risk factors. Finally, the more the cardiovascular risk factors, the higher the probability of suffering a myocardial infarction. The model showed good data fit: χ^2^(85) = 3617.87, *p* < 0.001; RMSEA = 0.030, 90% CI [0.029, 0.031]; CFI = 0.933; SRMR = 0.044. The RMSEA and SRMR are well below the 0.08 cut-off point, while the CFI is over 0.90 and very close to 0.95.

The standardized parameter estimates are presented in Figure 1. Age and gender had no relevant effects on the psychosocial factors. On the contrary, psychosocial factors had a substantial impact on frailty status. Overall, the four psychosocial factors were able to predict 50.3% of the variability of frailty status. The main predictors of frailty were well-being (β = −0.44, *p* < 0.01) and depression (β = 0.35, *p* < 0.01), while the effect of social connectedness was significant but low (β = −0.05, *p* < 0.05), and the effect of loneliness was not statistically significant (β = 0.01, *p* > 0.05). Frailty had a moderate effect on cardiovascular risk factors (β = 0.39, *p* < 0.01); this explained 15.5% of the variability of cardiovascular risk factors. In the end, cardiovascular risk factors increased the likelihood of having a myocardial infarction (β = 0.52). A total of 27.2% of the variability of myocardial infarction was associated with the cardiovascular risk factors.

Regarding the correlations among the antecedents, only age and gender were significantly related to each other, but with a very low value (r = −0.019, *p* < 0.01). The psychosocial factors were also correlated, however, for ease of understanding, the associations are not shown in Figure 1. The correlations that were significant, and moderate to large, were: loneliness with depression (r = 0.40, *p* < 0.01); loneliness with well-being (r = −0.47, *p* < 0.01); and depression with well-being (r = −0.46, *p* < 0.01).

## 4. Discussion

This work is intended as a contribution to the prediction of the occurrence of myocardial infarction from a holistic and processual approach. Regarding the holistic condition, to approach study of CVD, we should assess psychological risk factors and, simultaneously, the role of frailty [40,41]. In addition, the processual aspect reveals that there are variables to control, including some socio-demographics but also psychosocial factors that can trigger frailty in older adults. Indeed, frailty is a debilitating state that may exacerbate cardiovascular risk factors which may, in turn, improve cardiovascular outcomes. Finally, the accumulation of cardiovascular risk factors increases the likelihood of suffering myocardial infarction.

The model provides evidence on the important effects of social connectedness, depression, and psychological quality of life or well-being on frailty; factors which, in turn, indirectly affect cardiovascular risk factors. This processual model, ultimately, may predict 27% of the occurrence of myocardial infarction. The mechanism by which psychosocial factors increase the risk of myocardial infarction is likely multifactorial, although the precise pathophysiological factors remain to be elucidated. Experimental studies have shown that social disruption could lead to exacerbated coronary atherosclerosis and endothelial dysfunction, and connections between psychosocial variables and vascular function, inflammation, increased blood clotting, and reduced fibrinolysis have also been established [49]. Additional potential factors include increased hypothalamic–pituitary axis activity, increased sympathetic outflow, or modified behaviors that induce insulin resistance and central obesity [48]. The relationship between inflammation and depression has also been documented [73,74,75], sharing underlying pathophysiological aspects with physical frailty and CVD.

The biopsychosocial approach in CVD is not something new. Previous studies have mentioned it. However, only a few studies have tested it using complex theoretical models, often limiting themselves to more traditional ones. By 1980, Engel et al. [76] had already emphasized the importance of addressing mental health to prevent myocardial infarction. Borrell-Carrió et al. [77] suggested that this approach should guide clinical care and serve as a general practical guide. Lastly, Lurie et al. [29] evaluated the role of perceived social support in predicting the development of frailty following myocardial infarction. Our results reinforce those of this study, which concluded that below-average family income, poor self-rated health, and higher depression scores were all associated with frailty in a cohort of myocardial infarction survivors.

With this empirical research, we have clarified the negligible effect of gender in this process and connected more pieces in the picture puzzle of cardiovascular health, starting with the predictive capacity of an indicator of socioeconomic status on psychological aspects such as depression and loneliness, but not limited to this relationship. Our results support previous findings that depression increases the mortality and morbidity of cardiovascular disease [78], such as those of the American Heart Association, which concluded that depression should be considered a risk factor for poor prognosis among patients with acute coronary syndrome [79]. One surprising finding is that loneliness was not a statistically significant predictor in the model. Loneliness in older people has been associated in multiple studies with increased frailty [40,41] and with increased cardiac risk [80,81]. A plausible explanation for why loneliness is not a significant predictor in our study lies in the limitations of the scale used to measure loneliness. Since we used the short version of the three-item UCLA scale, we assessed loneliness as a composite factor like other studies [82]. However, other authors have expressed doubt about the scale’s unidimensionality [83], arguing that this scale cannot be used as a single factor because it does not assess a general experience of loneliness [84]. Future investigations could attempt to replicate these results using a less controversial measure of loneliness to clarify whether the limitations of the measure may be affecting the results of our research.

Our findings also have practical implications, such as the potential benefits of easily applicable interventions to achieve improvements in frailty [85] at the same time as preventing cardiac problems. By addressing these interconnected factors, interventions aimed at enhancing psychosocial dimensions and mitigating frailty could hold the potential to reduce cardiovascular risk, specifically myocardial infarction. Interventions that focus on improving psychological health can lead to reduced stress, anxiety, and depression. This, in turn, can positively impact cardiovascular health by reducing inflammation and promoting healthier lifestyles. Considering that frailty increases the likelihood of CVD, early detection is clinically relevant to provide a window for appropriate interventions [14]. Detection and prevention of frailty could also modulate the process of inflammation and thus prevent or delay the onset of CVD [17]. Studies provide evidence that diet and regular moderate physical activity reduce inflammation and frailty and improve cardiovascular health [86]. Comprehensive geriatric assessments are key for understanding increased vulnerability and designing personalized plans, encompassing physical and psychosocial approaches addressing underlying causes. Advancing precision medicine for older adults, including innovative assessments for common mechanisms like inflammation, is crucial and should adopt a biopsychosocial approach. Emerging geroscience, featuring anti-inflammatory, senolytic, and other mechanistic aging therapies, has the potential to transform care, directly influencing frailty reduction efforts of this research [16].

In addition, to ensure the success of these interventions, they should address potential barriers to participation for older people, including architectural barriers and difficulties with access to transportation [87].

This study’s strengths include the use of a large sample size, validated scales, and the use of multi-country European representative data. However, this study also has limitations. First, its cross-sectional nature precludes conclusions about direction of causation. The data are based on self-reported data, which could be affected by the self-reporting bias and issues related to common methods variance. Second, SHARE follows processes to ensure data reliability and validity such as interviewer, quality control of the interviews, and the use of CAPI to follow a semi-structured interview. However, interviewers have different characteristics that may be causing interviewer effect [88]; this should be considered when interpreting the results. Also, the influences of social desirability cannot be absolutely excluded.

Third, another aspect to consider as a limitation is the modification we have made to the EURO-D depressive symptomatology scale. Although the scale has shown good reliability for this study (α = 0.66, ω = 0.67), modification of the original scale may be altering the results. However, removing the fatigue and lack of appetite items in the EURO-D was necessary to avoid overlap with the frailty measure that also includes them. Future studies that use data where the measures do not overlap could investigate whether the results are replicated. Finally, there is a need to consider the non-inclusion of other variables that can have potential influence on this context due to the omission of its measurement in the SHARE protocol, for example, spirituality, which seems to play a key role in the adaptation of patients with cardiovascular risk and could enhance psychological health and reduce psychological disorders [89]. Also, further research could additionally improve the predictive power and the understanding of the process attending other variables. For example, including a more comprehensive examination of cardiovascular health and biological factors or the use of drugs such as antidepressants. The side effects of most antidepressant drugs are a problem for people at risk of cardiovascular disease and, therefore, it is necessary to assess their coexistence [90]. Also, we focus on social aspects such as loneliness, QoL, and social connectedness. However, other variables, such as social participation, should be addressed in future studies since they seem to have a negative relationship with hypertension [91].

## 5. Conclusions

In conclusion, studying the biopsychosocial model predicting myocardial infarction is crucial for advancing our knowledge of this complex condition and developing effective strategies for prevention, early detection, and patient care. A holistic approach focused on myocardial infarction should consider the interconnectedness of psychological well-being, depression, social isolation, and frailty, leading to more comprehensive and personalized approaches to managing cardiovascular health. Strategies related to physical activity and physical and mental lifestyles could be strategies that would have an impact, but future studies in this line are needed to assess whether interventions in mental health and frailty also improve cardiovascular health. Collaboration among healthcare professionals involves sharing information, jointly formulating treatment plans, mutual education, holding team meetings, and co-monitoring patients. This approach enables a comprehensive, personalized, and effective strategy for addressing patients’ mental, physical, and cardiovascular health.

Public health policies should be oriented towards community health that takes care of mental health and socialization. In addition, particularly for older people, special action regarding frailty is needed. These carefully designed actions could aim to promote cardiovascular health and prevent myocardial infarction. The evolving landscape of precision medicine regarding biomarkers of inflammation and study of the underlying mechanisms, along with community-focused initiatives, presents transformative opportunities for personalized older adult care and public health strategies.

## Figures and Tables

**Table 1:** Means, standard deviations, or percentages of all the observed variables in the model.

Variable	Mean or Percentage (Min–Max)	SD
Age	70.40 (50–103)	9.33
Mobility	1.74 (1–4)	1.10
Handgrip strength	31.92 (1–85)	11.28
Loneliness	3.99 (3–9)	1.44
Social connectedness	1.98 (0–4)	0.90
Economic status	2.77 (1–4)	1.01
Quality of life	37.29 (12–48)	6.32
Depressive symptomatology	2.01 (0–10)	1.92
Gender	Male = 42.6% Female = 57.4%	
Myocardial infarction	No= 86.9% Yes = 13.1%	
Hypertension	No = 54% Yes = 46%	
High cholesterol	No = 74.8% Yes = 25.2%	
Diabetes	No = 85.2% Yes = 14.8%	
Slowness	No = 87.2% Yes = 12.8%	
Appetite	No = 90.4% Yes = 9.6%	
Fatigue	No = 63.9% Yes = 36.1%	

## Data Availability

The data that support the findings of this study are available at the SHARE Research Data Center to the entire research community free of charge (www.share-project.org). Restrictions apply to the availability of these data, which were used under license for the current study and, thus, are not publicly available. Data are, however, available from the authors upon reasonable request and with permission of the SHARE Project (https://share-eric.eu/data/data-access).
